# D2H2: diabetes data and hypothesis hub

**DOI:** 10.1093/bioadv/vbad178

**Published:** 2023-12-04

**Authors:** Giacomo B Marino, Nasheath Ahmed, Zhuorui Xie, Kathleen M Jagodnik, Jason Han, Daniel J B Clarke, Alexander Lachmann, Mark P Keller, Alan D Attie, Avi Ma’ayan

**Affiliations:** Department of Pharmacological Sciences, Mount Sinai Center for Bioinformatics, Icahn School of Medicine at Mount Sinai, New York, NY 10029, United States; Department of Pharmacological Sciences, Mount Sinai Center for Bioinformatics, Icahn School of Medicine at Mount Sinai, New York, NY 10029, United States; Department of Pharmacological Sciences, Mount Sinai Center for Bioinformatics, Icahn School of Medicine at Mount Sinai, New York, NY 10029, United States; Department of Pharmacological Sciences, Mount Sinai Center for Bioinformatics, Icahn School of Medicine at Mount Sinai, New York, NY 10029, United States; Department of Pharmacological Sciences, Mount Sinai Center for Bioinformatics, Icahn School of Medicine at Mount Sinai, New York, NY 10029, United States; Department of Pharmacological Sciences, Mount Sinai Center for Bioinformatics, Icahn School of Medicine at Mount Sinai, New York, NY 10029, United States; Department of Pharmacological Sciences, Mount Sinai Center for Bioinformatics, Icahn School of Medicine at Mount Sinai, New York, NY 10029, United States; Department of Biochemistry, University of Wisconsin, Madison, WI 53706, United States; Department of Biochemistry, University of Wisconsin, Madison, WI 53706, United States; Department of Pharmacological Sciences, Mount Sinai Center for Bioinformatics, Icahn School of Medicine at Mount Sinai, New York, NY 10029, United States

## Abstract

**Motivation:**

There is a rapid growth in the production of omics datasets collected by the diabetes research community. However, such published data are underutilized for knowledge discovery. To make bioinformatics tools and published omics datasets from the diabetes field more accessible to biomedical researchers, we developed the Diabetes Data and Hypothesis Hub (D2H2).

**Results:**

D2H2 contains hundreds of high-quality curated transcriptomics datasets relevant to diabetes, accessible via a user-friendly web-based portal. The collected and processed datasets are curated from the Gene Expression Omnibus (GEO). Each curated study has a dedicated page that provides data visualization, differential gene expression analysis, and single-gene queries. To enable the investigation of these curated datasets and to provide easy access to bioinformatics tools that serve gene and gene set-related knowledge, we developed the D2H2 chatbot. Utilizing GPT, we prompt users to enter free text about their data analysis needs. Parsing the user prompt, together with specifying information about all D2H2 available tools and workflows, we answer user queries by invoking the most relevant tools via the tools’ API. D2H2 also has a hypotheses generation module where gene sets are randomly selected from the bulk RNA-seq precomputed signatures. We then find highly overlapping gene sets extracted from publications listed in PubMed Central with abstract dissimilarity. With the help of GPT, we speculate about a possible explanation of the high overlap between the gene sets. Overall, D2H2 is a platform that provides a suite of bioinformatics tools and curated transcriptomics datasets for hypothesis generation.

**Availability and implementation:**

D2H2 is available at: https://d2h2.maayanlab.cloud/ and the source code is available from GitHub at https://github.com/MaayanLab/D2H2-site under the CC BY-NC 4.0 license.

## 1 Introduction

Several web portals have been established to explore gene expression and other omics data from studies of diabetes and related metabolic disorders. For instance, diabetes-related gene expression and clinical phenotypes can be searched in the Diabetes Database (Keller *et al.* 2008). This database provides users with the ability to search for the expression of a gene, a gene set, or genes associated with a specific pathway in key tissues such as pancreatic islets and adipose as well as their obesity status, strain, and age. The T2D Knowledge Portal (KP) (Costanzo *et al.* 2023) hosts a variety of genetic information linked to type 2 diabetes, and it can be searched by gene, variant, genomic region, or phenotype. Additionally, T2D-KP offers a variety of bioinformatic tools that are integrated into the portal or hosted elsewhere. dkNET (Whetzel *et al.* 2015) is another central diabetes resource that directs users to bioinformatic tools, data, and other digital objects to enable data-driven diabetes research. dkNET specializes in indexing resources with identifiers. Websites that serve a collection of tools often just list them with the ability to filter or search by keywords to find the most relevant tools. These websites serve a purpose but are not all that useful for biologists seeking to analyze their data, integrate their data with public data, or form hypotheses from published data. Thus, it is advantageous to provide users with alternative methods for identifying the tools that are most relevant to their research questions, and the tools that can handle the data types that they aim to analyze. Recent emergence of Large Language Models (LLMs) coupled to tools and APIs (Liang *et al.* 2023, Shen *et al.* 2023) presents an opportunity to better present, access, and make available bioinformatics tools tailored to user needs (Lobentanzer and Saez-Rodriguez 2023). The Diabetes Data and Hypothesis Hub (D2H2) is a knowledge portal that contains hundreds of curated datasets, integrated tools to query single genes and gene sets, and a catalog of diabetes-related resources. By utilizing the OpenAI LLM application programming interface (API), D2H2 implements a chatbot that can answer gene and gene set-related queries as well as identify studies of interest available in a highly processed manner on the D2H2 site. In addition, by randomly selecting a gene set from the collection of D2H2 precomputed bulk RNA-seq signatures, and then querying this signature against gene sets extracted from supporting materials of publications, D2H2 produces hypotheses about the function of the randomly selected gene set. Hence, D2H2 facilitates the construction of data-driven hypotheses for the diabetes research community.

## 2 Methods

### 2.1 Curated datasets

The diabetes-related curated bulk and single-cell RNA-seq and microarray studies in D2H2 are sourced from the Gene Expression Omnibus (GEO) (Clough and Barrett 2016). When available, gene counts for these studies were obtained from ARCHS4 (Lachmann *et al.* 2018). The metadata for each study was manually formatted into tables with the samples and their corresponding conditions grouped for each study. Gene counts and metadata for bulk and single-cell (sc) RNA-seq studies are formatted as tab-separated TSV files and stored in an Amazon Web Services (AWS) Simple Storage Service (S3) bucket. For scRNA-seq studies, cluster identification is computed using the Leiden algorithm (Traag *et al.* 2019), and dimensionality reduction is performed with principal component analysis (PCA), t-distributed stochastic neighbor embedding (t-SNE) (Maaten and Hinton 2008), and Uniform Manifold Approximation and Projection (UMAP) (McInnes *et al.* 2018) using the Python package scanpy (Wolf *et al.* 2018). The final data matrix for each study is stored with its raw and normalized values, cluster labels, and dimensionality reduction coordinates as an Anndata object in AWS S3 to enable efficient access. Studies were additionally manually categorized by tissue, cell type, and disease with standardized identifiers sourced from the BRENDA Tissue Ontology (BTO) (Gremse *et al.* 2011) and the Human Disease Ontology (DO) (Schriml *et al.* 2022), respectively.

### 2.2 The gene expression in T2D transcriptomics signatures Appyter

The single-gene expression in type 2 diabetes (T2D) search engine is implemented as an Appyter (Clarke *et al.* 2021). An Appyter is a lightweight interactive independent bioinformatics tool that can be created from a Jupyter Notebook. The T2D Appyter provides a volcano plot visualization of the expression levels of a given gene in various type 2 diabetes transcriptomics signatures. Users enter a single gene symbol as input, and the output is a volcano plot that visualizes each T2D signature by gene-specific *P*-value (*y*-axis) and fold change (*x*-axis). The Appyter is available directly from D2H2 but also at https://appyters.maayanlab.cloud/#/Gene_Expression_T2D_Signatures.

### 2.3 Resources and tools integrated within D2H2

The D2H2 portal and its chatbot service integrate information from a variety of bioinformatics tools and databases. Some of these resources include GeneRanger (Marino *et al.* 2023), ARCHS4 (Lachmann *et al.* 2018), Enrichr (Chen *et al.* 2013), MGI-MP (Eppig 2017), Appyters (Clarke *et al.* 2021), GWAS catalog (Buniello *et al.* 2019), KEA3 (Kuleshov *et al.* 2021), ChEA3 (Keenan *et al.* 2019), and SigCom LINCS (Evangelista *et al.* 2022). The currently available tools (*n* = 14) and their data sources are described in Table 1. Gene and gene set analysis tools are also available directly from the D2H2 portal on dedicated web pages titled “Single Gene Queries” and “Gene Set Queries.”

**Table 1.** Inputs and outputs for each process currently available to the D2H2 chatbot and their data sources.

Input	Output	Data source
**Gene**	Diabetes signatures	GEO (Clough and Barrett 2016)
**Gene**	Expression	GeneRanger (Marino *et al.* 2023), ARCHS4 (Lachmann *et al.* 2018), GTEx (GTEx Consortium 2013, Jiang *et al.* 2020), Tabula Sapiens (Tabula Sapiens *et al.* 2022), CCLE (Ghandi *et al.* 2019, Nusinow *et al.* 2020), HPM (Kim *et al.* 2014), HPA (Thul and Lindskog, 2018)
**Gene**	Perturbations	Appyters (Clarke *et al.* 2021), GEO (Clough and Barrett 2016), L1000 (Subramanian *et al.* 2017)
**Gene**	Transcription factors	Enrichr (Chen *et al.* 2013)
**Gene**	Human GWAS traits	GWAS Catalog (Buniello *et al.* 2019)
**Gene**	Gene correlations	ARCHS4 (Lachmann *et al.* 2018)
**Gene**	Knockout phenotypes	MGI (Eppig 2017)
**Gene Set**	Diabetes signatures	GEO (Clough and Barrett 2016), Enrichr (Chen *et al.* 2013)
**Gene Set**	Enrichment	Enrichr (Chen *et al.* 2013), Rummagene (Clarke *et al.* 2023)
**Gene Set**	Transcription factors	ChEA3 (Keenan *et al.* 2019)
**Gene Set**	Kinases	KEA3 (Kuleshov *et al.* 2021)
**Gene Set**	L1000 small molecules and gene KOs	SigCom LINCS (Evangelista *et al.* 2022)
**Term**	Gene set	Enrichr (Chen *et al.* 2013), Rummagene (Clarke *et al.* 2023), Geneshot (Lachmann *et al.* 2019)
**Study metadata**	Studies	GEO (Clough and Barrett, 2016)

### 2.4 Process definitions

All the tools available from the D2H2 portal are defined in one JSON file (processes.json). Currently, these tools can accept two types of inputs: a [Gene], [GeneSet], [Term], or [Study Metadata]. For each input, there are designated output types, for example, [Traits], [Signatures], or [Expression]. Each output type has several attributes that are used to properly select, execute, and render the results from the selected tool. These include, for example, a description for selecting the tool; a JavaScript function to retrieve and render the desired result; a list of required parameters needed by the tool to perform the analysis; text for the user to describe the returned results; and a question that asks the user for additional input when options and parameters are missing. By integrating this information into one JSON file, additional tools can be added to expand the chatbot’s functionality. In addition, the same framework should work using other LLM services. Benchmarking such services could be achieved by scoring the correct selections of responses manually.

### 2.5 Interactions with the OpenAI GPT API

**Figure 1.**
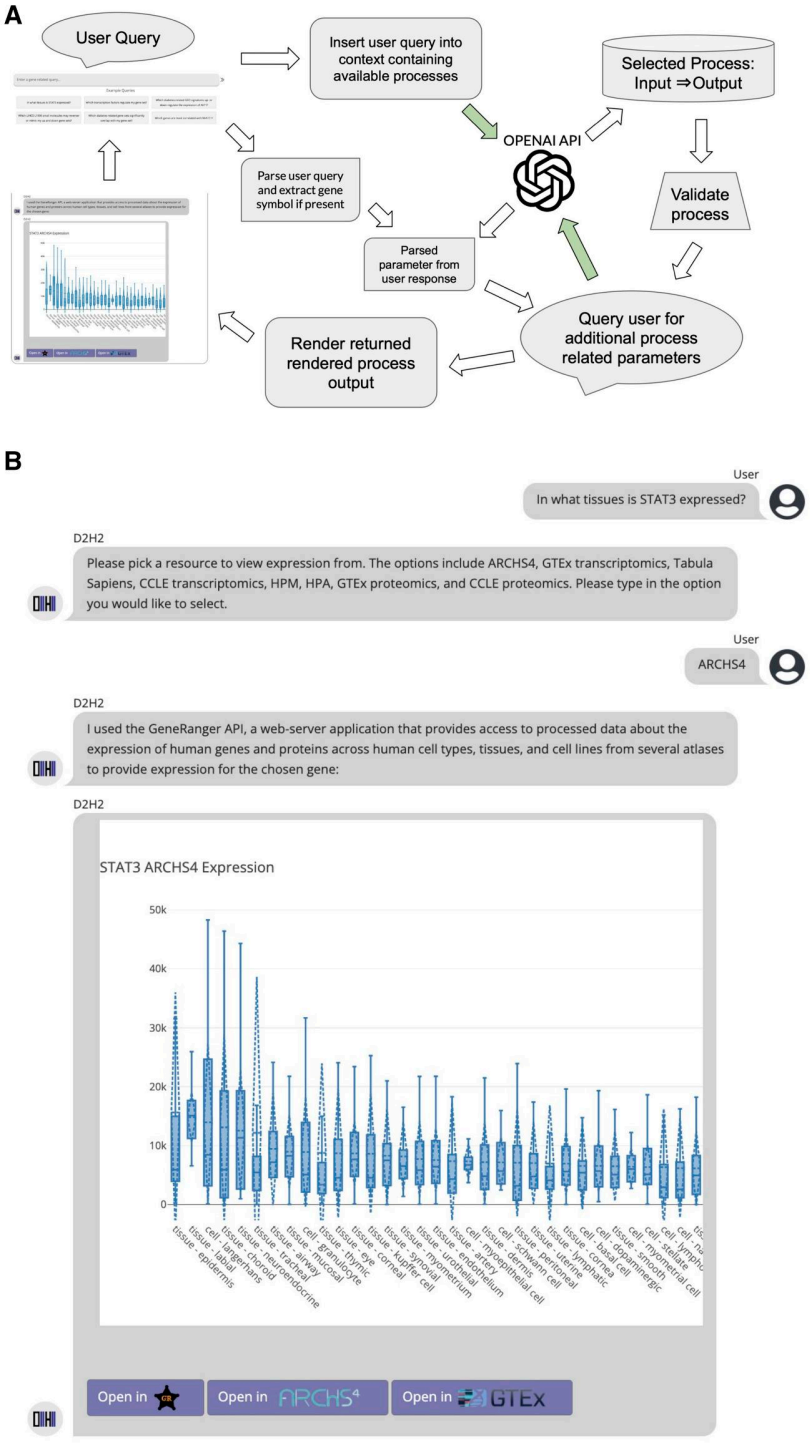
The D2H2 chatbot. (A) The user query is first sent to the OpenAI GPT API along with context that directs it to select a process. The process is validated based on a JSON file defining the implemented processes. The user is then queried for additional parameters if required for the chosen process. Once all parameters have been set, the result is rendered and sent to the chat where the user may then ask for another query. (B) Example chat sequence for the process [Gene] to [Expression].

When the user first enters a query, or selects an example query, this text is sent to the OpenAI API where it is inserted into a predefined prompt (Fig. 1). The chatbot first checks if the query’s input type aligns with one of the implemented types: [Gene], [GeneSet], [Term] or [Study Metadata]. It then selects from the available tools for that input based on the descriptions defined in the JSON file. It additionally instructs GPT that its response must only include the input and output types, and no other text, descriptions, or reasoning. The ChatCompletion class of the OpenAI python package (v0.27.5) is used to send the prompt to the “gpt-4” model with max_tokens set to 20 and temperature set to 0 to minimize the unpredictability of the model’s response. The response is parsed and validated based on the tools defined in the processes.json JSON file. If a returned tool is undefined, the chatbot will notify the user that it could not understand its query. If the user asks about a query with an input type not yet implemented, they will be presented with a list of numbered options to choose from. If all parameters are satisfied for a given tool based on the user query such as: “Which diabetes-related GEO signatures up or downregulate the expression of AKT1?”, then the JavaScript function associated with the tool will be called, and the output rendered in the chat interface. If additional parameters are required by the tool, such as specifying the atlas for providing expression levels across tissues and cell types, the user is queried again to enter their parameter selection based on the predefined question and a list of options given to the chatbot from the JSON file. The user’s response is then sent back to the OpenAI API with the user selections, and then the model is instructed to return the exact string from the options list that corresponds to the user’s response. If the response does not resemble any of the options, the model is instructed to respond “None”, and the user will be queried for another response to supply the missing parameter. This design enables the D2H2 chatbot to use an LLM to process user responses while mitigating the risks associated with hallucinations and inaccurate or inappropriate responses.

### 2.6 GPT-enabled hypothesis generation

Each day, a random, non-repeating, precomputed signature from D2H2 is submitted to Rummagene (Clarke *et al.* 2023), and the top 100 returned terms are ranked by abstract dissimilarity calculated using the scikit-learn Python package (Pedregosa *et al.* 2011). The term with the least similar abstract is selected and submitted to GPT-4 to form a hypothesis about the possible connection between the gene sets given both of their abstracts. All generated hypotheses are available on the Hypotheses page of D2H2. Users can also submit a gene set and a corresponding abstract to rank the top enriched terms from Rummagene by abstract dissimilarity. Terms that rank within the top five least similar abstracts are made available to be submitted to GPT-4 to automatically form hypotheses.

### 2.7 Full stack deployment technologies

The D2H2 portal is a Flask-based application running in a Docker virtual machine. It is served on a cluster managed with Kubernetes. The front end of the application and its styling are implemented with JavaScript, JQuery, Bootstrap, and HTML. The styling of the D2H2 chatbot utilizes the DaisyUI library.

## 3 Results

The D2H2 landing page features an LLM-powered chatbot that enables users to access a collection of tools via free text queries. The landing page of the portal provides a chatbot interface with nine example queries that may assist users with composing the types of queries D2H2 can handle. For example, a user may want to explore which tissues and cell types highly express a specific gene. D2H2 can answer this query by obtaining expression profiles for the gene from a variety of databases including ARCHS4 (Lachmann *et al.* 2018), GTEx (GTEx Consortium 2013), and Tabula Sapiens (Tabula Sapiens *et al.* 2022) using the GeneRanger API (Marino *et al.* 2023) (Fig. 1). Additionally, the chatbot can operate in a continuous fashion such that a user can ask several questions about a gene or gene set and have all this information provided on a single page. The single gene and gene set queries utilize a variety of resources to provide relevant information to the user. For single-gene queries, in addition to expression data served from GeneRanger (Marino *et al.* 2023), users may query transcription factors that regulate the gene from Enrichr (Chen *et al.* 2013), mouse knockout (KO) phenotypes from MGI-MP (Eppig 2017), drug perturbations that maximally increase or decrease the expression of the gene from GEO (Clough and Barrett 2016) and L1000 (Subramanian *et al.* 2017) through accessing two Appyters (Clarke *et al.* 2021), gene–gene correlations from ARCHS4 (Lachmann *et al.* 2018), and traits from the GWAS catalog (Buniello *et al.* 2019). For gene set queries, users may view enriched terms through Enrichr (Chen *et al.* 2013), transcription factors from ChEA3 (Keenan *et al.* 2019), kinases from KEA3 (Kuleshov *et al.* 2021), and L1000 small molecule perturbations and gene KOs from SigCom LINCS (Evangelista *et al.* 2022). Diabetes signatures computed from manually curated studies from GEO (Clough and Barrett 2016) can be queried with a single gene or a gene set. These results are linked to the individual study landing pages so the expression of the queried gene or gene set can be more thoroughly investigated. For users searching for gene sets associated with specific terms, the chatbot can query three different resources including Enrichr (Chen *et al.* 2013) to extract curated gene sets, Rummagene (Clarke *et al.* 2023) to search for gene sets extracted from the supplementary materials of PubMed Central publications, or Geneshot (Lachmann *et al.* 2019) which can create a gene set based on literature co-mentions with any PubMed search term. The chatbot additionally enables users to query D2H2 studies with free text, returning study tables with accompanying metadata. Overall, the D2H2 chatbot can provide users access to a collection of single gene and gene set analysis tools, providing a variety of outputs, while also enabling users to extract gene sets using several tools, or from precomputed differential expression analysis of queried studies.

The D2H2 website also currently (December 2023) hosts 162 human and 176 mouse transcriptomics datasets available to investigate via dedicated pages for each study. These pages contain interactive reports that provide uniform reanalysis of each study. The bulk RNA-seq and microarray study reports use a different template compared with the dedicated pages created for the scRNA-seq studies. Human and mouse studies are listed in searchable tables that include descriptions, sample count, cell-type/tissue, disease, and the corresponding publication. Users can change the species by using a toggle switch, and the table of listed studies can be filtered by standardized disease and tissue terms, enabling more focused browsing. Importantly, the tables of curated studies have a button that links to the specific study’s landing page. On the study landing page, a customized box plot viewer is implemented to view the expression of a single gene across conditions of the study. The conditions and corresponding samples shown in the boxplot can be toggled on or off. Users may view differential expression results precomputed with limma (Ritchie *et al.* 2015), or compute the differential expression across two conditions using either DESeq2 (Love *et al.* 2014) through the PyDESeq2 Python package (Muzellec *et al.* 2022) or the Characteristic Direction method (Clark *et al.* 2014). Precomputed differential expression is also available via the chatbot interface when querying for specific studies. The results from the differential expression analysis can be downloaded and submitted to Enrichr (Chen *et al.* 2013), Enrichr-KG (Evangelista *et al.* 2023), or the gene set queries page of D2H2. Single genes from the table may also be submitted to the single-gene queries page of D2H2. PCA, t-SNE (Maaten and Hinton 2008), and UMAP (McInnes *et al.* 2018) plots are available for each study report page. For bulk RNA-seq studies, users may submit the data to the Bulk RNA-seq Analysis Pipeline Appyter, which includes additional analyses and visualizations.

For example, if a user is interested in bulk RNA-seq studies conducted in mice brain, with a focus on type 2 diabetes, they can ask the D2H2 chatbot to return studies that meet this criterion, or filter the table of the studies by disease, tissue, and species. This search would assist the user to identify a study that examined the health benefits of co-administration of glucagon-like peptide 1 and peptide YY3–36 given to obese and diabetic mice (GSE160802) (Supplementary Fig. S1A) (Boland *et al.* 2022). A user can then investigate the expression of single genes across the study conditions. For instance, searching for the gene Stat3, we observe elevated expression in the brainstem and paraventricular nucleus of the hypothalamus when the mice are treated with the peptides compared to the vehicle control (Supplementary Fig. S1B). Performing differential expression with DESeq2 (Supplementary Fig. S1C), we can submit the top 250 upregulated genes with an adjusted *P*-value <0.05 to Enrichr and find that many enriched terms are associated with cholesterol metabolism, including Cholesterol Metabolism WP4718 from WikiPathway (Slenter *et al.* 2018). In previous work, it has been observed that diabetic insulin-deficient mice lack cholesterol in the hypothalamus due to a lack of expression of SREBP-2, an important regulator of cholesterol metabolism (Suzuki *et al.* 2010). SREBP-2 is contained in the significantly upregulated gene set due to the treatment, and it is also a member of many annotated cholesterol-related gene sets, suggesting that two-peptide treatment may increase SREBP-2 activity, and in turn enhances cholesterol metabolism in the brainstem of diabetic mice.

The Hypotheses page of D2H2 features automated GPT-generated hypotheses. To form these hypotheses, D2H2 gene sets created from the collection of bulk RNA-seq studies are queried against more than 600 000 gene sets extracted from the supporting materials of research publications listed in PubMed Central (PMC) using Rummagene (Clarke *et al.* 2023). Then a study is chosen that has high overlap with the D2H2 gene set and a low abstract similarity. The two abstracts, as well as the gene set names, are passed to GPT-4 for hypothesis generation. The LLM model is prompted to provide a possible explanation of why the studies with such dissimilar abstracts have such similar gene sets. The top of the page features an explanation of this feature and how the hypotheses are generated. The main section features the current daily hypothesis with relevant information such as the significance of the overlap, the PMC article title, and a link to the GSE study page on D2H2 (Supplementary Fig. S3). Additionally, this page features a module that enables users to submit their own gene set and an associated abstract to receive results from Rummagene of ranked highly overlapping gene sets with low abstract similarity. The top five entries are available to submit to GPT-4 for hypothesis generation.

The Help page of D2H2 details the various features of the site and how to navigate them. The curated studies in D2H2 as well as the processed signatures are available for download from the D2H2 Downloads page. The D2H2 Resources page provides a searchable table with various diabetes-related resources, associated publications, funding information, and keywords. Additionally, a D2H2 Twitter bot is implemented. This bot queries PubMed each day for identifying diabetes-related gene-centric publications, and tweets about these new publications with links to analyze the genes mentioned in each study. The D2H2 About page contains links to the many resources and tools that are integrated across the site as well as provides pie-chart visualizations of various metadata attributes. A majority of the studies on the site are from RNA-seq experiments compared to microarray (Supplementary Fig. S2A) and the most common disease type is diabetes (non-specific), followed by gene knockouts, type 2 diabetes, and type 1 diabetes (Supplementary Fig. S2B). The most common tissues associated with studies on D2H2 are the pancreas, adipose, and liver (Supplementary Fig. S2C) and the most common cell types of interest for the scRNA studies included pancreatic islets and beta cells (Supplementary Fig. S2D). In general, however, D2H2 contains some studies that are not just relevant to diabetes specifically, but also to other related metabolic disorders. Importantly, the D2H2 site also provides a form that provide an opportunity for users to suggest missing datasets.

**Figure 2.**
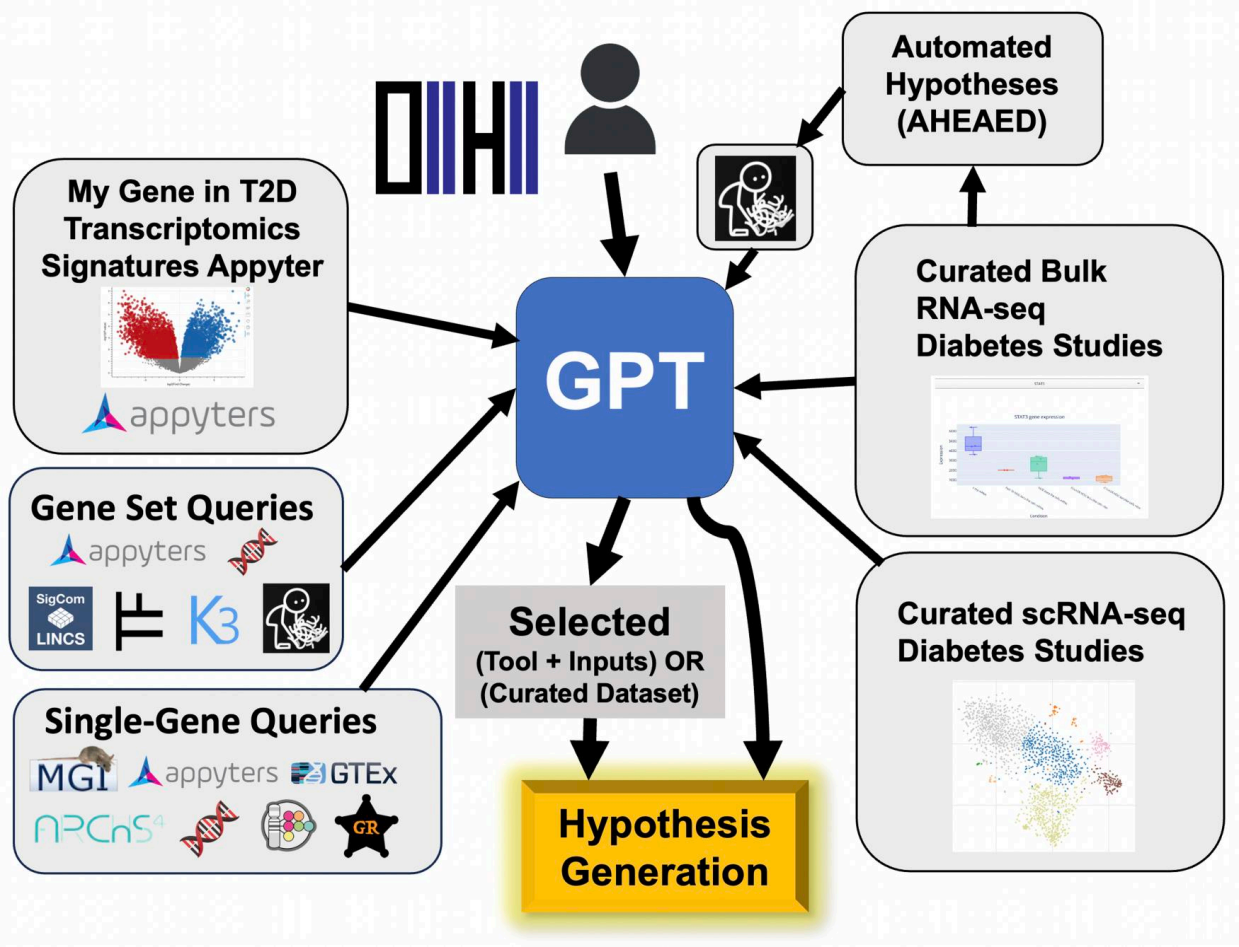
Workflow diagram of the content served on the D2H2 portal. The D2H2 portal provides means to submit a single gene, or a gene set, query through the D2H2 chatbot or on dedicated pages. It then processes these queries with a variety of bioinformatics tools and databases to provide knowledge and answer relevant specific questions. The portal also randomly selects D2H2 gene sets from the bulk RNA-seq computed signatures, and automatically forms hypotheses about these signatures by finding highly overlapping gene sets with abstract dissimilarity. Bolded terms in the workflow diagram represent different tabs on the D2H2 portal, and the logos surrounding each term symbolize the tools and databases that are utilized on those web pages. AHEAED, Automated Hypothesis Each and Every Day.

Overall, D2H2 serves as an integrator of a variety of tools, resources, and datasets related to diabetes biomedical research (Fig. 2). It lowers the barrier to entry for researchers who are not computationally oriented. The site also offers analysis of a wide variety of studies manually curated from GEO. These studies are processed to be easily searchable, and differential expression can be directly investigated with many tools available from the D2H2 site. The processed diabetes signatures provide users with a way to access and query these studies given their own single gene, gene set, or gene signature inputs. In the future, we plan to continue to process and add more diabetes-related studies into D2H2, and continually update the D2H2 chatbot with more and better tools and add other capabilities. We will also plan to develop a knowledge graph that will connect diabetes researchers based on their co-authored publications and the genes that they study.

## Supplementary Material

vbad178_Supplementary_Data
