# Supplementary material for: D2H2: diabetes data and hypothesis hub
Source: Bioinform Adv. 2023 Dec 4;3(1):vbad178. doi: 10.1093/bioadv/vbad178 (PMC10723036; doi:10.1093/bioadv/vbad178)
Supplement: vbad178_Supplementary_Data [file vbad178_supplementary_data.pdf]

ONLINE SUPPORTING MATERIALS

D2H2: Diabetes and Data and Hypothesis Hub

Giacomo B. Marino[1], Nasheath Ahmed[1], Zhuorui Xie[1], Kathleen M. Jagodnik[1], Jason Han[1], Daniel J. B. Clarke[1], Alexander Lachmann[1], Mark P. Keller[2], Alan D. Attie[2], and Avi Ma'ayan[1,*]

[1]Department of Pharmacological Sciences, Mount Sinai Center for Bioinformatics, Icahn School of Medicine at Mount Sinai, New York, NY; [2]Department of Biochemistry, University of Wisconsin, Madison, WI.

*To whom correspondence should be addressed.

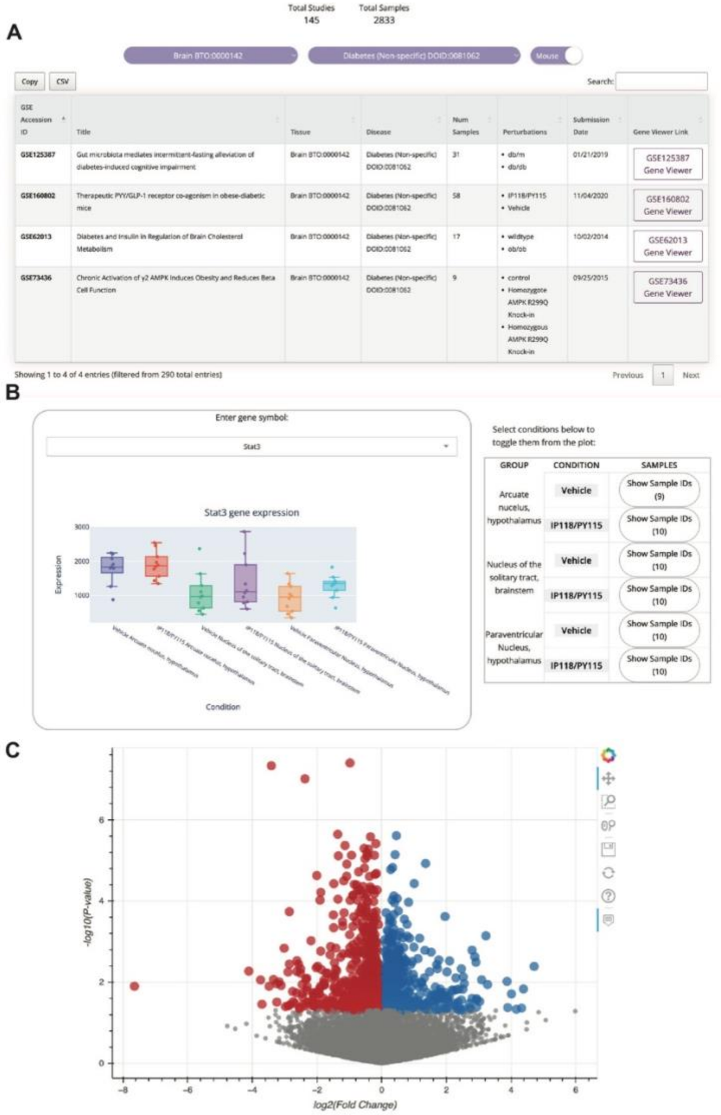

Figure S1. Investigating processed GEO study workflow. A. The user can filter the table of studies based on species, tissue, disease type, and a free text query. **B.** The study viewer page presents a customized boxplot viewer wherein the expression of a single gene can be visualized across selected conditions. **C.** Differential expression computed with DESeq2 for Vehicle, Nucleus of the solitary tract, brainstem vs. IP118/PY115, Nucleus of the solitary tract, brainstem.

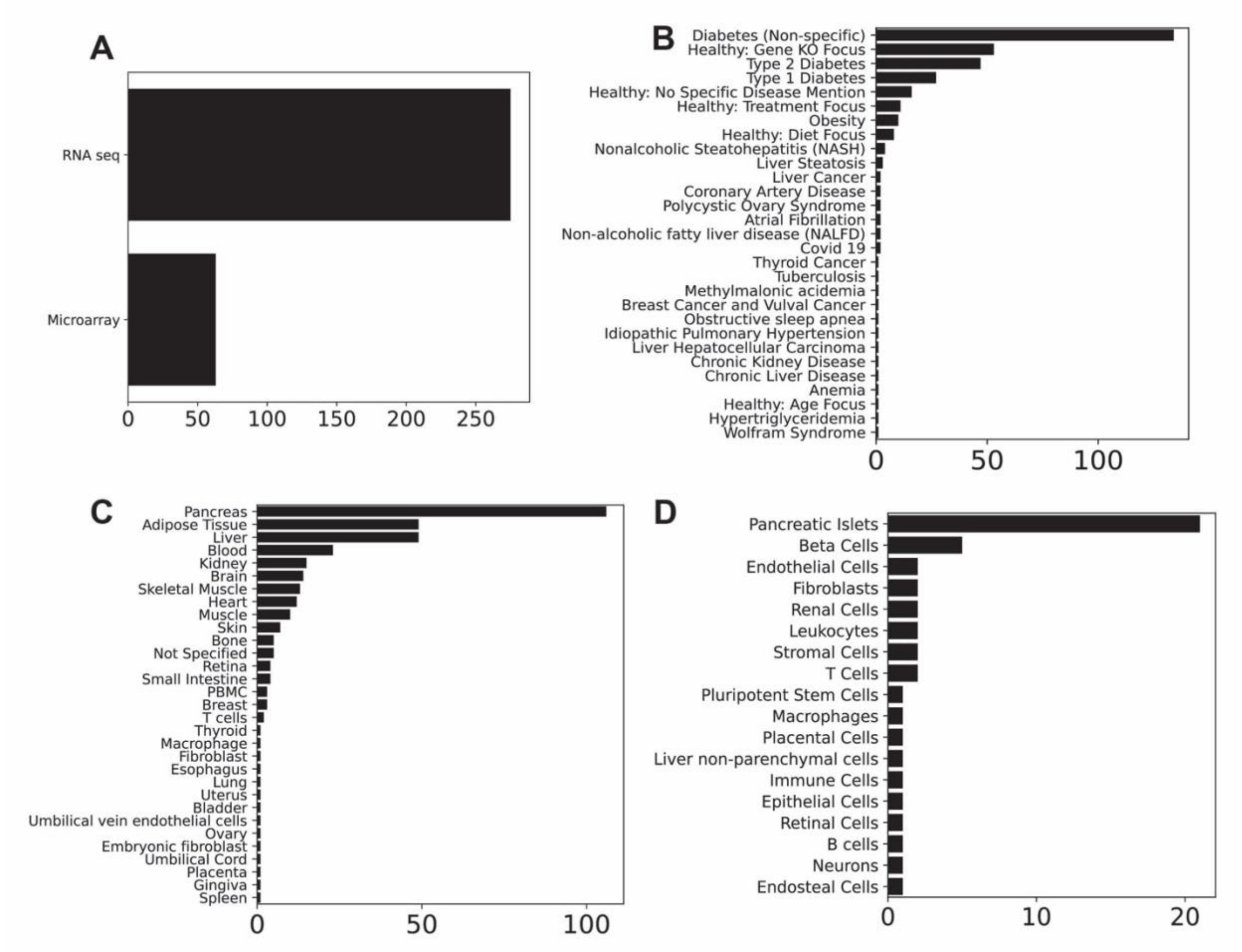

Figure S2. Manually Curated metadata statistics for Bulk RNA-seq, microarray and scRNA-seq studies. A. Assay types across all D2H2 studies. **B.** Disease types across all studies. **C.** Tissue and cell types for bulk RNA-seq and microarray studies. **D.** Cell types of focus for scRNA-seq studies.

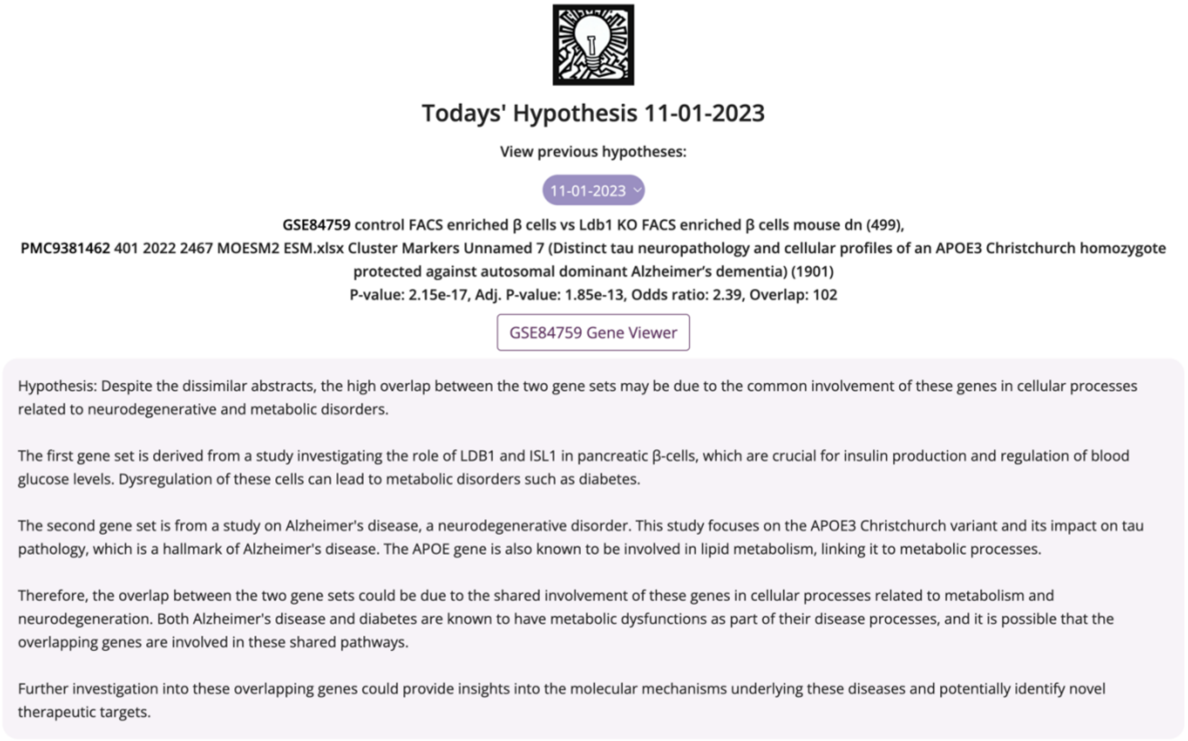

Figure S3. D2H2 Automated Hypothesis Each and Every Day (AHEAED). Daily GPT-4 generated hypothesis generated from a significantly overlapping D2H2 signature and extracted gene set from PubMed Central through utilizing corresponding abstracts and gene set names.
